# Pulsatile gonadotropin-releasing hormone: clinical applications of a physiologic paradigm

**DOI:** 10.1016/j.xfre.2023.01.007

**Published:** 2023-02-02

**Authors:** Marco Filicori

**Affiliations:** Reproductive Endocrinology Center, University of Bologna, Bologna, Italy

**Keywords:** GnRH, pulsatile, LH, gonadotropins, ovulation induction

## Abstract

Gonadotropin-releasing hormone (GnRH) is a fundamental driver of human reproduction. A pulsatile pattern of GnRH secretion is essential to achieve pituitary stimulation, gonadotropin secretion, and normal gonadal function. Pulsatile GnRH administration is used to treat anovulation and male hypogonadotropic hypogonadism. Pulsatile GnRH ovulation induction is effective and safe because it allows to avoid ovarian hyperstimulation syndrome and reduce the occurrence of multiple pregnancies. This physiology-inspired therapeutic tool has also permitted to elucidate several pathophysiologic features of human reproductive disorders.

**Essential Points**

• Discovery of gonadotropin-releasing hormone and the pulsatile nature of its secretion have profoundly affected the understanding of the hypothalamic-pituitary-gonadal axis physiology.
• Changes in pulsatile LH secretion across the normal human menstrual cycle are critical to insure normal folliculogenesis, ovulation, and corpus luteum function.
• Pulsatile gonadotropin-releasing hormone administration is a safe and effective way to treat anovulation-related infertility and male hypogonadotropic hypogonadism.

Control of human gonadal function depends on pituitary gonadotropin secretion, although this relationship is not a simple constant stimulatory action by luteinizing hormone (LH) and follicle-stimulating hormone (FSH). Since it became possible to measure serum gonadotropin levels with radioimmunoassay, it was recognized that FSH and LH act in a different but complementary way to achieve folliculogenesis, ovulation, and conception. In addition, gonadal hormones modulate gonadotropin secretion in different fashions across the menstrual cycle.

The prevalence of FSH secretion in the late luteal phase (LLP) and early follicular phase (EFP) causes ovarian follicular recruitment; the prevalence of LH secretion in the midfollicular phase (MFP) and late follicular phase (LFP) results in the selection of the dominant follicle; a relatively short and high burst of LH induces dominant follicle breakup, oocyte release, and corpus luteum (CL) formation; LH secretion, again, is capable of CL and endometrial support to provide an optimal environment for embryo implantation.

For several years, it was not clear how these physiologic fluctuations of gonadotropin secretion could be achieved in such a coordinated and perfectly timed fashion that guaranteed optimal reproductive function while maintaining the low multiple conception rate that is characteristic of the human species. Critical breakthroughs in this scientific area were the characterization and synthesis of gonadotropin-releasing hormone (GnRH) (1, 2) and the understanding of the critical role that the pattern of pulsatile GnRH, and hence, pulsatile LH, plays in the modulation of ovarian function (3).

## Spontaneous pulsatile gonadotropin secretion

**Figure 1:**
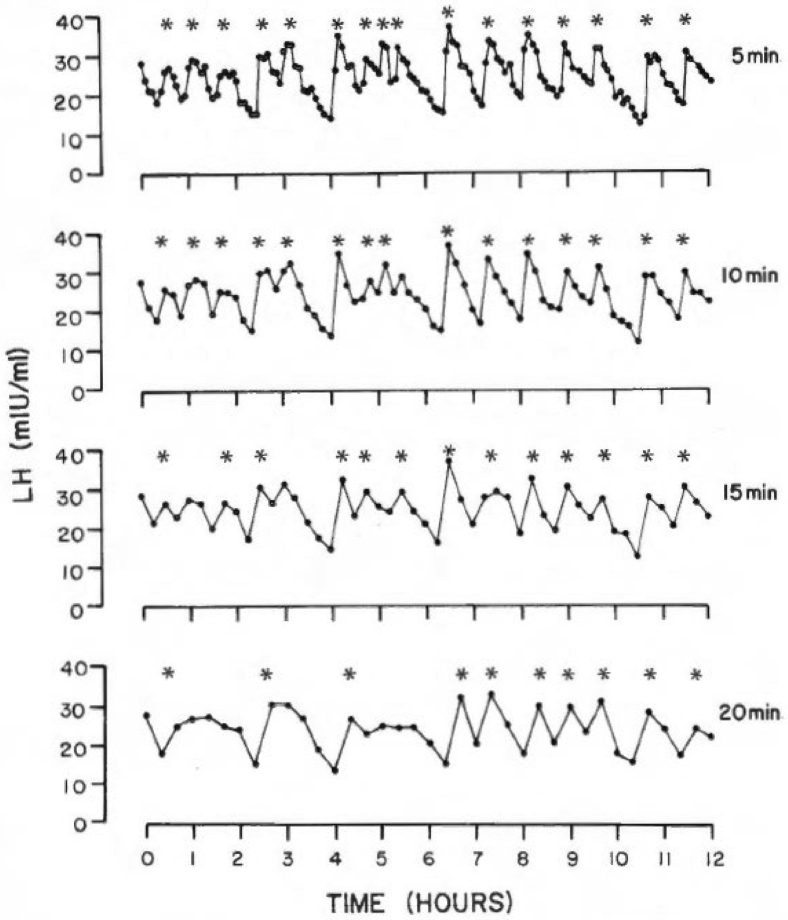
Luteinizing hormone (LH) measurements in blood samples obtained at 5-minute intervals for 12 consecutive hours in a patient with polycystic ovary syndrome. Data points were then progressively omitted to simulate blood sampling conducted at 10-, 15-, and 20-minute intervals. The asterisks indicate significant LH peaks identified by computer algorithm. (From Crowley et al. (6). Reprinted by permission of the publisher.)

Several studies conducted in the 1970s showed that pulsatile LH secretion changes across the human menstrual cycle, with faster pulses occurring in the follicular phase (FP) and slower, but wider, pulses in the luteal phase (LP) and until menses (4, 5). Nevertheless, these investigations were conducted with blood samples drawn at 20-minute intervals for a limited timespan (6–8 hours) that did not permit a complete characterization of this physiologic phenomenon. Thus, early on in our program, it became evident that a critical aspect for pulsatile LH secretion characterization was blood sampling frequency. When compared with the blood sampling frequency previously adopted, we showed that shortening blood sampling intervals to 10 minutes or less allowed to identify more LH peaks, thus improving the ability to detect most relevant short-term LH fluctuations (Fig. 1) (6). We also elected to expand the duration of these pulse analysis studies to a period to 24 consecutive hours. Although this approach yielded a more detailed and robust assessment of LH pulsatility, it also meant that with a 10-minute blood sampling procedure over 24 hours, 144 consecutive samples had to be obtained. Thus, we developed a blood sparing sampling procedure that limited the amount of each sample drawn to 3 mL of blood, without discarding any more blood and avoiding dilution from the saline drip needed to keep the intravenous (i.v.) line open. In this manner, we were able to limit the total amount of blood obtained at a total of 432 mL, as stipulated with the Medical Ethics Committee of Massachusetts General Hospital in Boston.

With these methodological approaches, we conducted the largest normative pulsatile LH study of the human spontaneous menstrual cycle so far published and were able to assess with far greater precision the normal pulsatile LH profiles in the spontaneous menstrual cycle of normal women (7). In the FP, the frequency of LH peaks progressively increased from 1 pulse every 94 minutes in the EFP to 1 every 71 minutes in the LFP but before the onset of the preovulatory LH surge, which was characterized by an outburst of even more rapid and large LH peaks. In the LP, conversely, the LH peaks significantly slowed down from 1 every 103 minutes in the early luteal phase to 1 every 216 minutes in the LLP. The LH pulse amplitude was markedly more elevated in the LP than in the FP. In a separate study (8), we also showed that the phenomenon of hormone pulsatility is not limited to the hypothalamic-pituitary system; in the LP, progesterone is also secreted in a pulsatile fashion by the ovary, and its peaks closely mirror pituitary LH secretion. In addition to its relevance for the understanding of CL physiology, this finding has important clinical implications because it shows that widely variable progesterone levels can occur within a few hours, thus limiting the usefulness of single progesterone determinations to assess CL function and viability. On the basis of these results, we were also able to adjust the regimens we used for pulsatile GnRH ovulation induction.

## Treatment with pulsatile GnRH

The discovery and synthesis of hypothalamic polypeptides that induced gonadotropin secretion (1, 2) raised the hope that more “physiologic” and, thus, more efficient and safe means of indirect ovarian stimulation could be achieved by eliciting endogenous gonadotropin secretion via GnRH administration. However, it soon became evident that a daily regimen GnRH administration, such as the one employed for exogenous gonadotropins, was not capable of achieving sustained secretion of pituitary gonadotropins and, thus, of gonadal function (9). Furthermore, infrequent GnRH administration regimens, far from achieving consistent gonadal stimulation, end up suppressing gonadotropin and gonadal steroid levels. Only intermittent (pulsatile) GnRH administration had the power of stimulating pituitary and gonadal function in males and females (3). Two clinical approaches for the management of gonadal function, thus, emerged: pulsatile GnRH administration for stimulation and GnRH agonists (and later antagonists) for suppression.

## Ovulation induction

Since the 1960s, it was shown that administration, at daily intervals, of exogenous gonadotropins (extracted from cadaver pituitaries or postmenopausal urine) was able to stimulate ovarian steroid secretion, the development of ovarian follicles, and ovulation (10); this latter objective was achieved with human chorionic gonadotropin (hCG) administration as a form of high-grade LH activity because LH could not be extracted in sufficient amounts from biologic material. Although highly effective in the treatment of infertility in anovulatory patients, this therapeutic modality was also associated with high rates of complications such as ovarian hyperstimulation syndrome (OHSS) and high-grade multiple pregnancies. At that time, exogenous gonadotropin treatment of anovulation was not easily controllable because of a lack of effective ovarian monitoring tools, such as pelvic ultrasound and rapid serum estradiol measurements.

**Figure 2:**
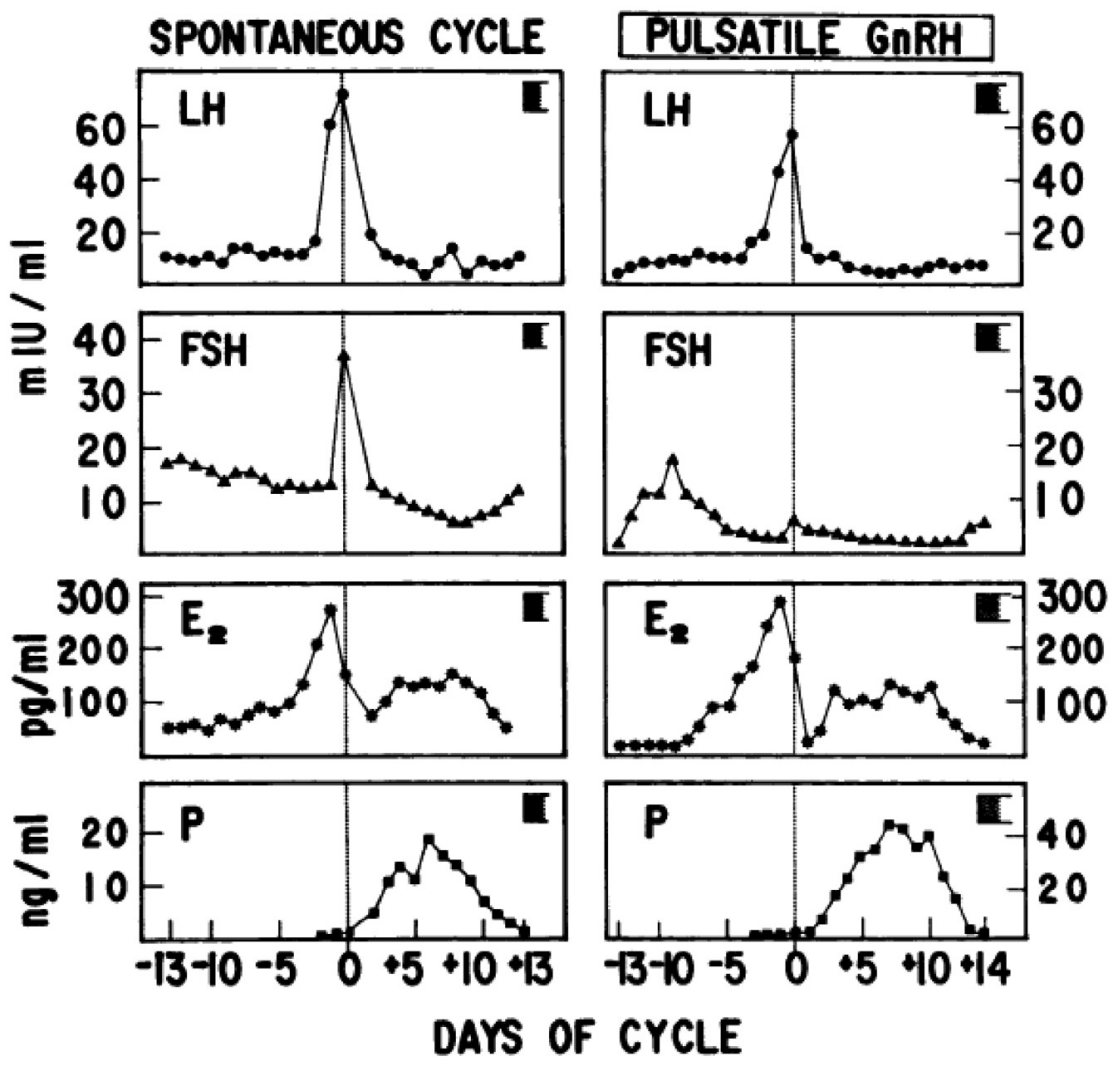
Daily gonadotropin and gonadal steroid levels throughout a normal spontaneous menstrual cycle (*left panels*) and during pulsatile gonadotropin-releasing hormone (GnRH) ovulation induction in a patient with hypothalamic primary amenorrhea (*right panels*). E_2_ = estradiol; FSH = follicle-stimulating hormone; LH = luteinizing hormone; P = progesterone. (From Filicori et al. (13). Reprinted by permission of the publisher.)

The characterization and synthesis of GnRH, a compound that stimulates gonadotropin secretion and, indirectly, gonadal function, raised the hope that more physiologic tools could become available. The demonstration by Knobil (3) that pulsatile GnRH was capable of consistently stimulating gonadotropin and gonadal function in subhuman primates was followed by the first publications in humans that proved that this treatment modality could be applied in humans and that it was capable of closely reproducing the normal reproductive hormone dynamics of the menstrual cycle (11) and achieve pregnancy (12). The critical endocrine features of pulsatile GnRH administration are most evident when this drug is administered to patients with profound forms of hypogonadotropic hypogonadism (e.g., Kallmann syndrome) that are characterized by a virtually complete absence of endogenous GnRH secretion and primary amenorrhea. In these subjects, the sole source of pituitary stimulation is represented by exogenously administered GnRH, without interference from endogenous GnRH. When such patients are carefully monitored during treatment with daily blood sampling for the measurement of gonadotropins and gonadal steroids, the following features emerge (Fig. 2) (13):

• An early but temporary increase in the LFP level in the EFP that stimulates ovarian follicle recruitment; thereafter, declining FSH secretion in the MFP of these cycles curtails further development of most follicles and prevents the ovulation of multiple oocytes that is the cause of multifetal pregnancies in gonadotropin ovulation induction.
• Low LH levels in the EFP that progressively increase across the FP; the resulting prevalence of LH over FSH in the MFP and LFP allows the dominant follicle (which more efficiently expresses granulosa cell LH receptors) to continue to grow and mature despite declining FSH levels.
• Progressive increments of serum estradiol levels that peak at physiologic levels of 300–500 pg/mL, compatible with the development of a single dominant follicle.
• A spontaneous preovulatory midcycle LH surge of physiologic length that occurs without modifications in exogenous GnRH dose and frequency or the administration of other hormones, such as hCG; this surge is likely caused by the positive feedback action of ovarian hormones (e.g., a modest progesterone increment) that takes place at maximum dominant follicle maturity.
• Excellent CL and endometrial support, as evidenced by physiologically elevated serum levels of progesterone and estradiol, and successful implantation. Furthermore, when discontinuation of pulsatile GnRH is preferred (e.g., so that the drug delivery device can be used to treat another patient), CL support can be achieved with low amounts of exogenous hCG, with no risk of OHSS.
• Even a mild increment of the serum FSH level in the last days of the LLP that, when pulsatile GnRH is not discontinued, starts follicular recruitment in the following treatment cycle.

To optimize the safety of the drug delivery system, we also improved the i.v. setup provided by the pharmaceutical company that manufactures the drug delivery device because it consisted of a thin i.v. catheter that could not be safely held in place by the dressing applied after catheter insertion. In a patient treated at a different clinic, such catheter became loose, was lost, and had to be surgically recovered from the patient’s venous circulation, fortunately without further complications. Thus, we chose to employ a peripheral i.v. catheter (Angiocath; Becton Dickinson and Company, Franklin Lakes, NJ), securely held in place with a Tegaderm transparent dressing (3M Health Care, Saint Paul, MN) that also allowed us to monitor proper placement and conditions of the i.v. insertion site. Furthermore, because the drug reservoir of pulsatile GnRH pumps does not need to be changed for several days (i.e., being substantially a closed system), the delivery system i.v. line is usually maintained in place for at least 10–14 days. We were concerned that the presence of an indwelling i.v. line for such a long period of time (traditional guidelines mandated that the insertion site of peripheral i.v. lines should be changed every 48–72 hours) could cause septic complications. Thus, we performed blood cultures and microbiologic controls on 230 catheter tips used in 38 female patients for at least 7 consecutive days. Only 2% of blood cultures turned positive, and none was associated with local or systemic signs of infection; none of the patients developed fever (14). Thus, this system proved to be effective and safer than other setups used for pulsatile GnRH administration; the i.v. line had to be reinserted only occasionally, when peripheral vein occlusions occurred.

## Frequency of pulsatile GnRH administration

Our administration rate for pulsatile GnRH was chosen on the basis of our studies of the physiologic frequency of LH pulses occurring in the FP of the menstrual cycle (7). The GnRH-induced pulse profile is important in women because it was found that even modest slowing of the hypothalamic-pituitary pulse generator was associated with anovulation (15).

Adherence to a physiology-based GnRH pulse frequency and dose administration regimen permitted to better standardize this treatment modality. For ovulation induction, published studies have employed a frequency of GnRH administration of every 60–120 minutes (in most cases every 90 minutes), whereas the GnRH dose has ranged between 2.5 and 20 μg/bolus; the higher GnRH dosages are usually employed when GnRH is administered subcutaneously (s.c.). We attempted to mimic more closely the frequency of GnRH-driven LH peaks that occur in the FP of the normal menstrual cycle (7). We found that the MFP and LFP LH peaks occur at close to 1-hour intervals; thus, we adapted this frequency of GnRH administration. Furthermore, we employed this model to determine whether slower LH pulse intervals per se could affect folliculogenesis, ovulation, and CL function (16).

**Figure 3:**
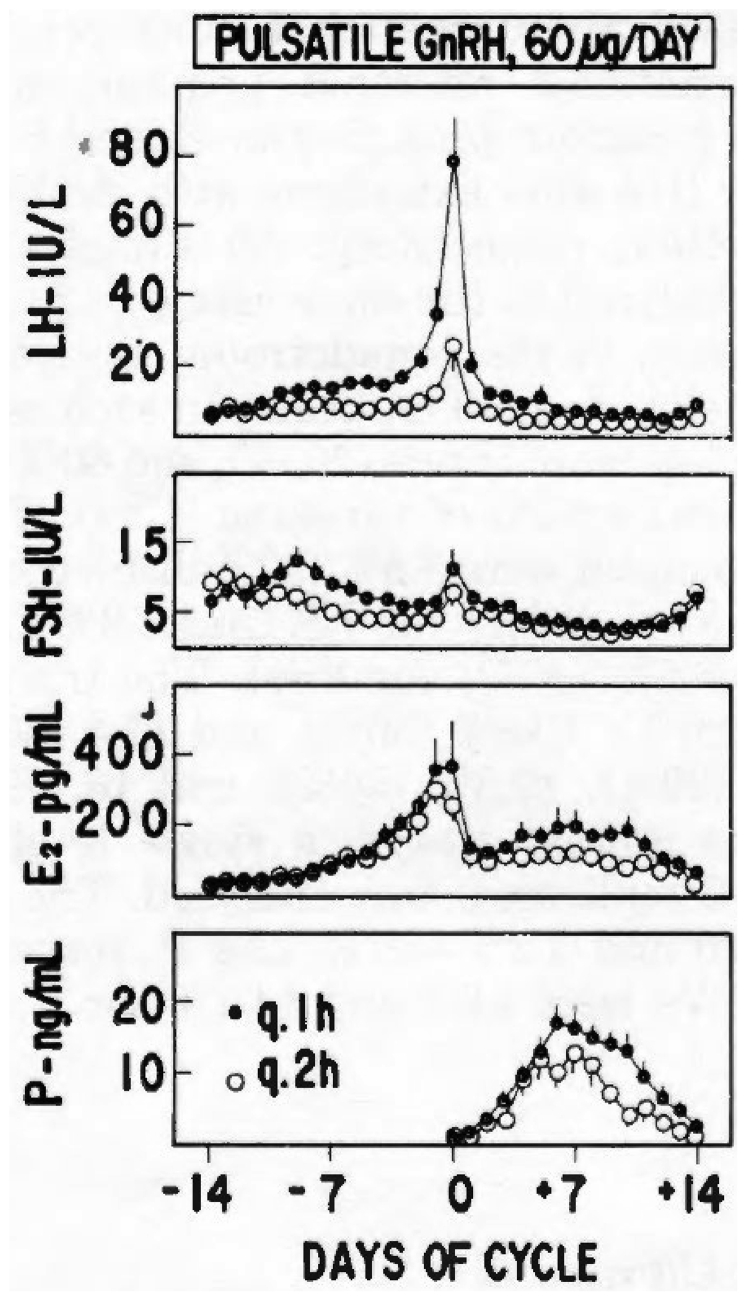
Daily gonadotropin and gonadal steroid levels (mean ± standard error) in patients with primary hypogonadotropic amenorrhea receiving 60 μg/day of GnRH at 60-minute intervals (closed circles) or 120-minute intervals (open circles). E = estradiol; FSH = follicle-stimulating hormone; LH = luteinizing hormone; P = progesterone. (From Filicori et al. (16). Reprinted by permission of the publisher.)

Although the mean LH and FSH levels in women with hypothalamic amenorrhea or oligomenorrhea are often within the normal range, anovulation is usually present. We speculated that the presence of an abnormal GnRH-driven LH pulse frequency could be the pathogenetic factor underlying deranged ovulation because LH peaks tend to be significantly slower in these patients than in the FP of normal women. Thus, we tested the potential effect of GnRH pulse frequency and dose variations in women with primary hypogonadotropic amenorrhea. These patients mostly have no spontaneous LH pulses in 12–24-hour frequent sampling measurements, suggesting an absent or quiescent endogenous GnRH secretion that represents the ideal ablation/replacement model to investigate the effect of exogenous GnRH administration, without interference from endogenously secreted GnRH. We then administered to these patients pulsatile GnRH at a dose of 2.5, 5.0, or 10 μg/bolus at 60- or 120-minute intervals, obtaining a total daily GnRH dose of 60 or 120 μg. With this protocol, we were able to demonstrate that a 60-minute GnRH administration frequency achieved ovulation in 89%–100% of cycles, whereas the slower 120-minute frequency (albeit with the same daily GnRH dose) only caused ovulation in 57%–81% of treatments. Anovulation appeared to be related to a lower and blunted midcycle LH surge; CL function was also affected because patients treated with a GnRH pulse frequency of 120 minute had lower serum LP estradiol and progesterone levels (Fig. 3). Thus, we confirmed that slower endogenous GnRH-induced LH pulsatility is a relevant pathogenetic mechanism in the development of anovulation. The reduced success rate of slower pulsatile GnRH administration can be partly corrected by the use of higher per pulse amounts of GnRH (e.g., 10 or 20 μg). A recent meta-analysis confirmed that optimal pulsatile GnRH administration is achieved through i.v. GnRH administered at 60–90-minute intervals (17). As to the route of pulsatile GnRH administration, although the s.c. route with LH boluses administered at 2-hour intervals provided appropriate ovarian response, the optimal outcome was achieved with i.v. GnRH administration.

## Pulsatile GnRH treatment in polycystic ovary syndrome

**Figure 4:**
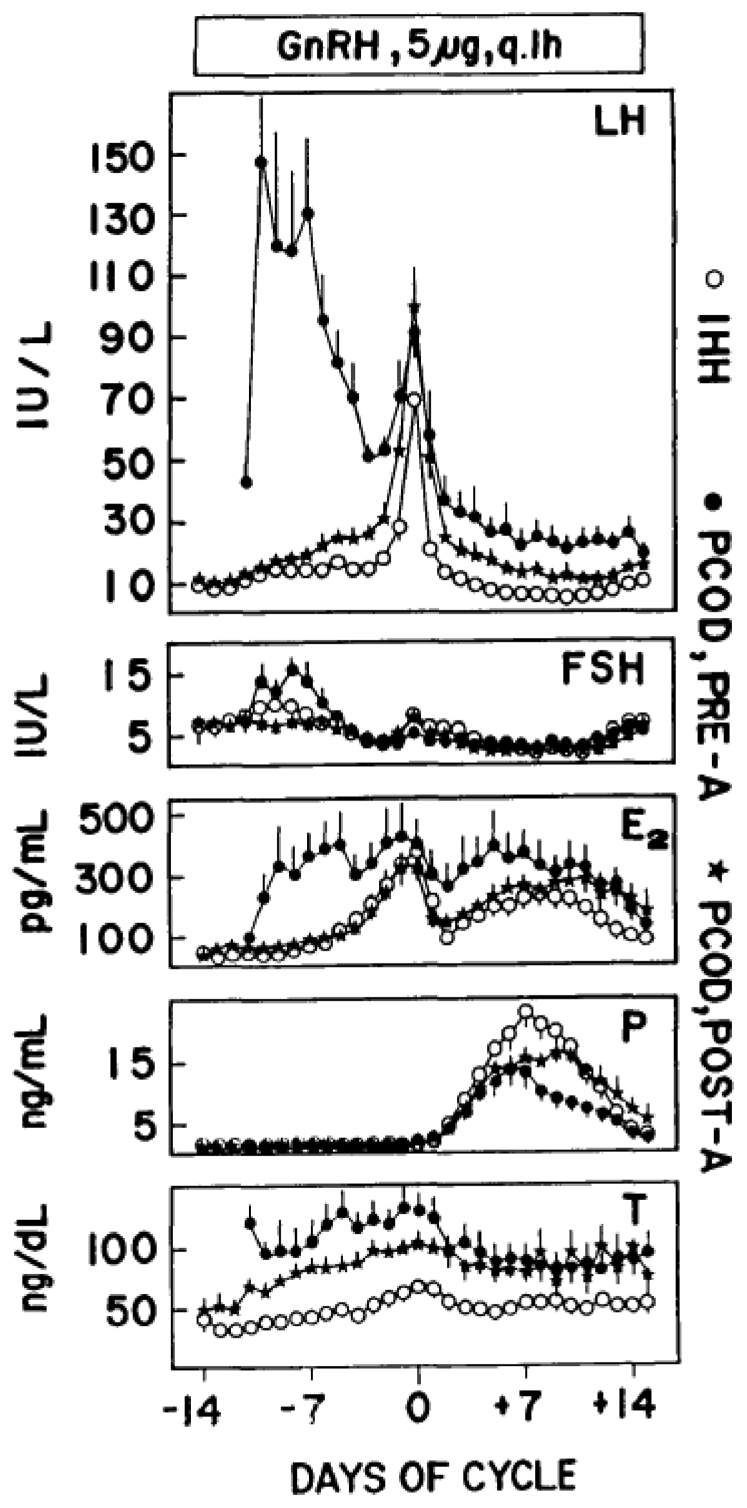
Daily gonadotropin and steroid levels (mean ± standard error) during pulsatile gonadotropin-releasing hormone (GnRH) administration (5 μg/bolus at 1-hour intervals) in the ovulatory cycles of patients with idiopathic hypogonadotropic hypogonadism (IHH) (O), polycystic ovary disease (PCOD) without GnRH agonist suppression (pre-A [•]), and PCOD after GnRH agonist suppression (post-A [∗]). Hormone data are centered around the midcycle gonadotropin surge. E = estradiol; FSH = follicle-stimulating hormone; LH = luteinizing hormone; P = progesterone. (From Filicori et al. (18). Reprinted by permission of the publisher.)

One group of anovulatory patients who were found to be relatively resistant to standard pulsatile GnRH administration is composed of women with polycystic ovary syndrome (PCOS) (18,19). In these patients, pituitary gonadotropin reserve is often markedly increased, so that, when pulsatile GnRH is administered, the serum LH and FSH levels dramatically increase, thus affecting menstrual cycle hormone dynamics, folliculogenesis, and ovulation; CL function may also be disrupted. These patients often fail to respond to pulsatile GnRH or, conversely, develop excessive folliculogenesis that may lead to multiple gestations (20). Although i.v. pulsatile GnRH appears to be more effective than that via the s.c. route, the real breakthrough came when we developed a pharmacologic regimen meant to correct excessive pituitary gonadotropin reserve by pretreating PCOS with a GnRH agonist for 8 weeks and starting pulsatile GnRH administration immediately thereafter (18). With this regimen, pituitary gonadotropin reserve was drastically reduced, the LH and FSH levels and secretory dynamics were much closer to the ones of the normal menstrual cycle and of hypogonadotropic women treated with pulsatile GnRH (Fig. 4), and a markedly improved clinical response was obtained, with higher ovulation and pregnancy rates. This regimen also reduced the rates of multiple conceptions in patients with PCOS. These findings were confirmed by our group in a follow-up study (21).

## Clinical outcome and complications

Several years ago, we reported the results of the largest series of pulsatile GnRH ovulation induction cycles published in a single study (22). We assessed treatment outcome in 600 consecutive cycles of pulsatile GnRH ovulation induction in 292 female patients with infertility with different reproductive disorders: idiopathic hypogonadotropic hypogonadism associated with primary or secondary amenorrhea; PCOS or multifollicular ovaries; and other forms of hyperandrogenic anovulation. Pulsatile GnRH was administered i.v. at dosages of 1.25–20.0 μg/bolus, at frequencies of 1 pulse every 30–120 minutes; most cycles were performed with regimens of 2.5–5.0 μg/GnRH bolus every 60–90 minutes. In 228 cycles of patients with excessive ovarian activity, pulsatile GnRH administration was preceded by 6–8 weeks of pituitary-gonadal suppression with a GnRH agonist (buserelin 300 μg s.c., twice a day).

We reported an overall ovulatory rate of 75%, being highest in patients with primary hypogonadotropic amenorrhea (83%) and lowest in patients with PCOS (65%). In this study, the overall pregnancy rate per ovulatory cycle was 23%, that is, similar to the mean pregnancy rate in spontaneous menstrual cycles (23). In terms of complications, moderate or severe OHSS did not occur in any of the 600 treatment cycles, also because hCG was never used to trigger ovulation. The multiple pregnancy rate in this study was 3.8%; however, all multiple pregnancies were low grade (twins and only 1 triplet), and only 1 multiple gestation (1.4%) of 69 pregnancies occurred in patients with low pituitary reserve, either spontaneous (primary hypogonadotropic amenorrhea) or induced by GnRH agonist before treatment, indicating that only when pulsatile GnRH hits a pituitary with an excessive FSH reserve the resulting high FSH secretion can cause multiple folliculogenesis that usually precedes multiple ovulation and conception. The overall miscarriage rates were relatively elevated at 30%; however, in this study, we included pregnancy demise occurring within 2 weeks from a positive pregnancy test, that is, before ultrasound confirmation (biochemical pregnancies).

## Pulsatile GnRH in hypogonadotropic males

Hypogonadotropic hypogonadism occurs less frequently in males than in females. Nevertheless, a similar impairment in the hypothalamic GnRH pulse generator represents the key pathogenetic feature of this condition in both sexes. Pulsatile GnRH/LH secretion is critical also for male reproductive function; however, their dynamics of secretion are relatively constant and not dependent on subtle gonadal hormone modulation as in females. Pulsatile GnRH administration proved to be an excellent therapeutic tool in males (24), although the long duration of treatment (several consecutive months or years) required to induce normal reproductive maturation and gametogenesis renders this approach more cumbersome than with exogenous gonadotropin or androgen administration. Nevertheless, it was shown that pulsatile GnRH administration appears to be faster than exogenous gonadotropins to induce and maintain spermatogenesis (25). Pulsatile GnRH administration in males is usually performed at dosages of 5–25 ng/kg of body weight of GnRH administered s.c. at 2-hour intervals. In this setting, the i.v. drug administration route is impractical because of the long duration of treatment. The per-bolus GnRH route may need to be increased up to 600 ng/kg when response to treatment appears to be suboptimal. Pulsatile GnRH treatment causes normalization of the profile of androgen secretion and testicular volume in the great majority of males with idiopathic hypogonadotropic hypogonadism, although pre-existing disorders, such as cryptorchidism, may negatively affect treatment outcome. Good levels of spermatogenesis can also be obtained in most of these patients.

In summary, pulsatile GnRH administration was found to be an extremely safe and effective tool to treat hypogonadotropic men and women and correct anovulation. Although pulsatile GnRH is no longer therapeutically available in several countries, this represents a missed opportunity to expand the therapeutic armamentarium available for these patients. Almost nonexistent complications, such as OHSS and multiple conceptions, and, thus, the reduced need for treatment monitoring make pulsatile GnRH an attractive alternative to exogenous gonadotropins for the management of human reproductive disorders.
